# Crystal structure of 2-(methyl­amino)­tropone

**DOI:** 10.1107/S2056989019009502

**Published:** 2019-07-09

**Authors:** Leandri Jansen van Vuuren, Hendrik G. Visser, Marietjie Schutte-Smith

**Affiliations:** a Department of Chemistry, PO Box 339, University of the Free State, Bloemfontein, 9301, South Africa

**Keywords:** crystal structure, 2-(methyl­amino)­tropone, tropolone

## Abstract

The title compound, 2-(methyl­amino)­cyclo­hepta-2,4,6-trien-1-one, C_8_H_9_NO, crystallizes in the monoclinic space group *P*2_1_/*c*, with three independent mol­ecules in the asymmetric unit. The planarity of the mol­ecules is indicated by planes fitted through the seven ring carbon atoms. Small deviations from the planes, with an extremal r.m.s. deviation of 0.0345 Å, are present. In complexes of transition metals with similar ligands, the large planar seven-membered aromatic rings have shown to improve the stability of the complex. Two types of hydrogen-bonding inter­actions, C—H⋯O and N—H⋯O, are observed, as well as bifurcation of these inter­actions. The N—H⋯O inter­actions link mol­ecules to form infinite chains. The packing of mol­ecules in the unit cell shows a pattern of overlapping aromatic rings, forming column-like formations. π–π inter­actions are observed between the overlapping aromatic rings at 3.4462 (19) Å from each other.

## Abstract 2

The title compound crystallizes in the monoclinic space group *P*2_1_/*c* with three independent mol­ecules in the asymmetric unit. Two types of hydrogen-bonding inter­actions, C—H⋯O and N—H⋯O, are observed, as well as bifurcation of these inter­actions. The N—H⋯O inter­actions link mol­ecules to form infinite chains. The packing of mol­ecules in the unit cell shows a pattern of overlapping aromatic rings, forming column-like formations. π–π inter­actions are observed between the overlapping aromatic rings.

## Chemical context   

Tropolone and other troponoids, non-benzenoid compounds, have great pharmacological potential (Guo *et al.*, 2019 ▸). They display a wide range of bioactivities, including anti­microbial (Saleh *et al.*, 1988 ▸), anti­viral (Tavis & Lomonosova, 2015 ▸) and anti­tumor (Ononye *et al.*, 2013 ▸) activities. Many tropolone-related compounds have proved to be possible anti­proliferative agents against a variety of cancer cell lines, including lung, prostate and T-cell malignancies (Liu & Yamauchi, 2006 ▸; Hsiao *et al.*, 2012 ▸). Tropolone has important medical applications in radiopharmacy (Nepveu *et al.*, 1993 ▸) and as catalyst precursor (Crous *et al.*, 2005 ▸; Roodt *et al.*, 2003 ▸).

Tropolone and its derivatives are versatile ligands used in inorganic and organometallic chemistry (Roesky, 2000 ▸; Dias *et al.*, 1995 ▸; Nozoe *et al.*, 1997 ▸; Schutte *et al.*, 2010 ▸; Steyl *et al.*, 2010 ▸). The carbonyl oxygen and vicinal coordinating substituent, specifically nitro­gen in this study, impart a metal-chelating ability to these types of ligands. The complexes of these ligands with first and second row transition elements have increased over the past few decades. The ligands of importance in this study and future work, namely 2-(alkyl­amino)­tropones and amino­troponimines, are *N*,*O* and *N*,*N*′ bidentate, monoanionic ligands containing a ten π-electron backbone (Roesky, 2000 ▸). The π-conjugated backbone is characteristic of these ligands (Nishinaga *et al.*, 2010 ▸). Considering the above-mentioned characteristics, tropolone could be considered analogous to the O-donor κ^2^-*O*,*O*′ acetyl­acetonate ligand (acac-*O*,*O*′). The tropolone bidentate ligand differs from the acac-*O*,*O*′ ligand in a few ways. Of importance to our study is the larger aromatic delocalization, which could afford greater polarizability. Tropolone is also more acidic than the acac-*O*,*O*′ ligand. The acidity of the *O*,*N* and *N*,*N*′ bidentate ligands used in our study and the effect thereof on the chelating ability could be compared to these *O*,*O*′ bidentate ligands described in the literature. The ligand–metal–ligand angle, better known as the ‘bite angle’, would also be smaller for a tropolone-derived complex, since it would form a five-membered metallocycle instead of a six-membered one as with acac-*O*,*O*′ (Bhalla *et al.*, 2005 ▸). This could show inter­esting steric and electronic influences at the metal centre and could be further compared to the steric and electronic studies conveyed on β-diketone moieties in similar metal complexes by Manicum *et al.* (2018 ▸). These ligands, including the title compound, 2-(methyl­amino)­tropone, will form part of the synthesis of water-soluble complexes of rhenium(I) tricarbonyl, gallium(III) and copper(II). Rhenium(I) (Schutte-Smith *et al.*, 2019 ▸), gallium(III) (Green & Welch, 1989 ▸) and copper(II) (Boschi *et al.*, 2018 ▸) are highly utilized radioisotopes in the radiopharmaceutical industry.

When designing diagnostic or therapeutic radiopharmaceuticals, certain mechanistic aspects are very important, as it is the basis on which some predictions are made regarding the *in vivo* behaviour. Kinetic studies, utilizing different techniques, are executed to determine the reaction mechanisms by which the proposed radiopharmaceutical complexes will form and react. Results of such studies are important in nuclear medicine as it gives indications regarding the *in vivo* stability, uptake and excretion as well as the pharmacokinetics of the compounds. Kinetic investigations by Schutte *et al.* (2011 ▸, 2012 ▸), Schutte-Smith *et al.* (2019 ▸) and Manicum *et al.* (2019 ▸) were done on rhenium(I) tricarbonyl tropolonato complexes with satisfying results and conclusions. In the study, methanol substitution was studied using entering nucleophiles in *fac*-[Re(Trop)(CO)_3_(MeOH)]. The kinetic study performed at high pressure indicated positive volumes of activation for all of the reactions studied. This was a clear indication towards a dissociative inter­change mechanism.

The application of these ligands in coordination chemistry could be further increased by adding electron donating or withdrawing moieties to the nitro­gen atom.
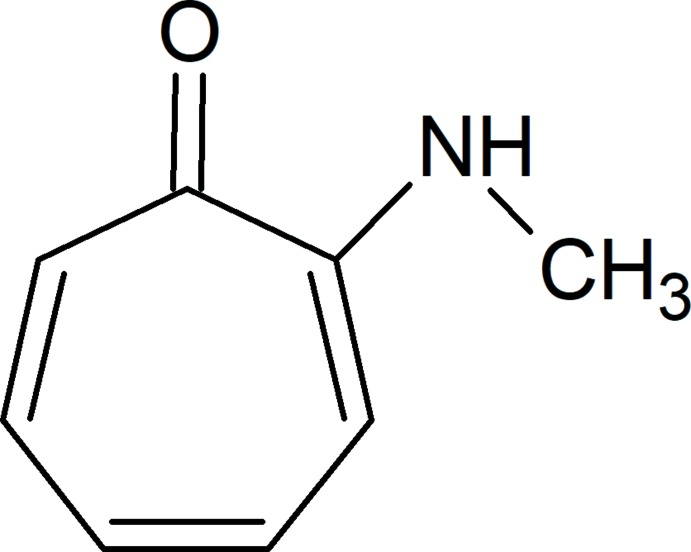


## Structural commentary   

**Figure 1:**
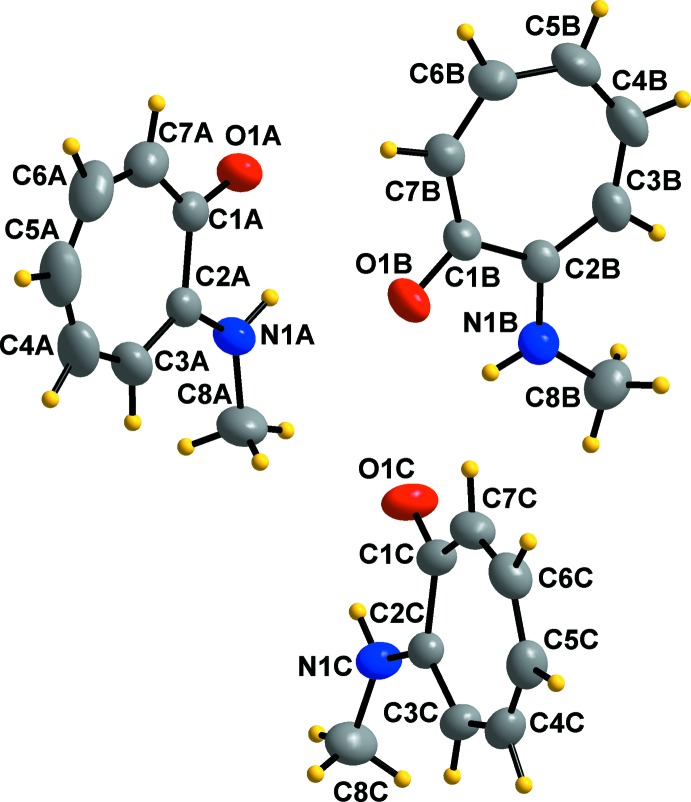
The mol­ecular structure of 2-(methyl­amino)­tropone, indicating the numbering scheme, with displacement ellipsoids drawn at the 50% probability level.

2-(Methyl­amino)­tropone crystallizes in the monoclinic *P*2_1_/*c* space group with three independent mol­ecules, *A*, *B* and *C*, in the asymmetric unit (Fig. 1 ▸). The bond distances and angles of the three mol­ecules agree well with each other and with those in similar structures (Barret *et al.*, 2014 ▸; Dwivedi *et al.*, 2016 ▸; Roesky & Bürgstein, 1999 ▸; Shimanouchi & Sasada, 1973 ▸; Siwatch *et al.*, 2011 ▸). The largest differences in bond distances are of the C8*B*—N*1B* [1.4470 (18) Å], N1*B*—C2*B* [1.3444 (17) Å] and O1*B*—C1*B* [1.2561 (15) Å] bonds with the corresponding bonds in 2-(*t*-butyl­amino)­tropone [1.472 (4) Å; Siwatch *et al.*, 2011 ▸], 2-(iso­propyl­amino)­tropone [1.330 (4) Å; Roesky & Bürgstein, 1999 ▸] and 2-(*t*-butyl­amino)­tropone [1.242 (4) Å; Siwatch *et al.*, 2011 ▸], respectively. Compared to the starting material mol­ecule, tropolone (Shimanouchi & Sasada, 1973 ▸), the O1*B*—C1*B* [1.2561 (15) Å] bond distance is slightly shorter than that of tropolone [1.261 (3) Å], both being in the range of normal C=O bond distance. The C2—N1—C8 bond angle in mol­ecules *A* [125.69 (13)°], *B* [125.27 (13)°] and *C* [125.07 (12)°] are slightly larger than the usual 120° for trigonal-planar bond angles, because of the steric influence of the methyl group. These angles are close to the same angle in 2-(benzyl­amino)­tropone [125.09 (12)°; Barret *et al.*, 2014 ▸]. This could be compared to the large angle in 2-(*t*-butyl­amino)­tropone [131.9 (2)°; Siwatch *et al.*, 2011 ▸], which deviates even more from 120° due to the highly steric tertiary butyl group. A plane fitted through the seven ring carbon atoms of the three mol­ecules in the asymmetric unit indicates that the mol­ecules are planar. The root-mean-square deviations of mol­ecules *A*, *B* and *C* from the planes are 0.0141 (12), 0.0261 (11) and 0.0345 (11) Å, respectively. The C8—N1—C2—C3 torsion angle, which involves the methyl group, differs for mol­ecule *A* [−0.8 (2)°], mol­ecule *B* [2.3 (2)°] and mol­ecule *C* [7.7 (2)°]. The small deviations from planarity could possibly be ascribed to the inter­molecular hydrogen-bonding inter­actions.

## Supra­molecular features   

**Figure 2:**
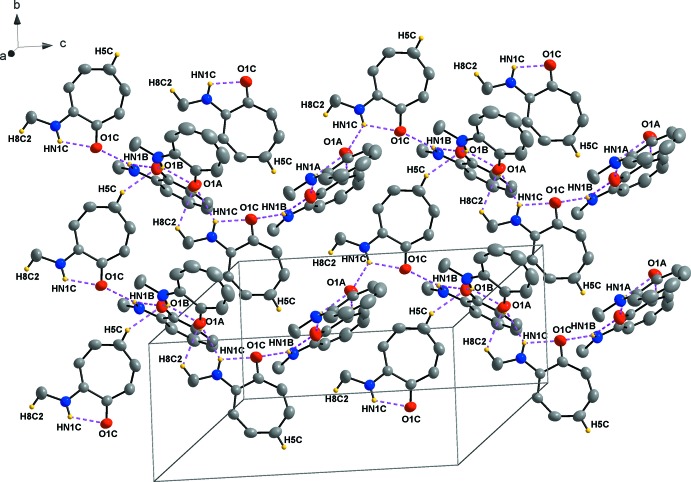
Hydrogen-bonding inter­actions (Table 1 ▸) and infinite chains along [001] in the unit cell.

**Figure 3:**
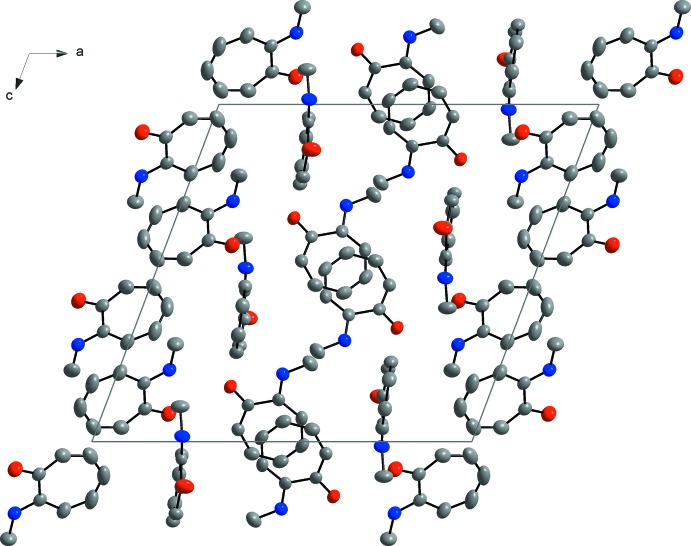
Packing of mol­ecules viewed perpendicular to the *ac* plane.

**Figure 4:**
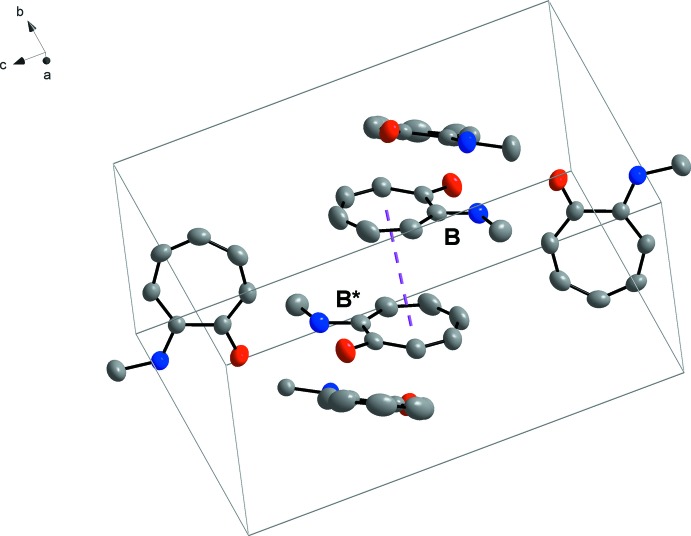
π–π inter­action (highlighted by the dashed line) between overlapping aromatic rings of mol­ecule *B*, where B and B* are related through inversion.

Nine hydrogen-bonding inter­actions, three C—H⋯O and six N—H⋯O, are observed (Table 1 ▸ and Fig. 2 ▸). Infinite chains are formed along [001]. These supra­molecular chains are formed through N—H⋯O inter­actions linking the mol­ecules together. As in the crystal structure of tropolone (Shimanouchi & Sasada, 1973 ▸), bifurcation of the hydrogen bonds take place. Bifurcation, also known as the over-coordination of a hydrogen bond, creates both inter- and intra­molecular branches, which might contribute to the stability of the structures. This is an inter­esting phenomenon seen in the orientation of water mol­ecules, where the distribution of acceptor hydrogen bonds, terminating at the lone pairs of the oxygen, is higher (Markovitch & Agmon, 2008 ▸). This forms over-coordinated oxygens and could also be seen in this crystal structure (Fig. 2 ▸). These inter­actions clearly contribute to the array of the mol­ecules in the asymmetric unit (Fig. 2 ▸). The mol­ecules show an inter­esting packing format in the unit cell. ‘Column’-like structures are formed by mol­ecule *B* packing in a head-to-tail pattern with the aromatic rings overlapping (Fig. 3 ▸). A π-inter­action is observed, with a perpendicular distance of 3.4462 (19) Å between the overlapping aromatic rings of two inversion-related *B* mol­ecules (Fig. 4 ▸). These π-inter­actions could not only possibly contribute to the packing format of the mol­ecules in the unit cell, but could also assist in the formation of one-dimensional infinite chains, as Wong *et al.* (2018 ▸) have found in water-soluble platinum (II) salts.

## Database survey   

A search of the Cambridge Structural Database (CSD, Version 5.40, update of February 2019; Groom *et al.*, 2016 ▸) using a C_7_H_5_ONH fragment yielded four hits of 2-(alkyl­amino)­tropones. These include 2-(iso­propyl­amino)­tropone (LIGVOM: Roesky & Bürgstein, 1999 ▸), 2-(benzyl­amino)­tropone (NOPRUH: Barret *et al.*, 2014 ▸), 2-(t-butyl­amino)­tropone (OZINUH: Siwatch *et al.*, 2011 ▸) and 2-(cyclohexyl­amino)­tropone (OTIMUB: Dwivedi *et al.*, 2016 ▸). Of the four structures, only the 2-(iso­propyl­amino)­tropone and the 2-(benzyl­amino)­tropone crystallize in the *P*2_1_/*c* space group.

## Synthesis and crystallization   

Tropolone (505 mg, 4.132 mmol) was dissolved in 20 mL of a 40% methyl­amine solution. The reaction mixture was stirred at room temperature for 7 d. The product was extracted three times with 30 mL of chloro­form, and the organic layer was washed with 50 mL of water. The organic layer was dried with Na_2_SO_4_ and the solvent removed under reduced pressure. A 46.03% yield (257.1 mg, 1.902 mmol) was obtained. Crystals suitable for single crystal X-ray diffraction data collection were obtained by recrystallization from hexane with slow evaporation. Yield: 0.2571 g, 46.03%. IR (cm^−1^): ν_NH_ = 3304, ν_CO_ = 1597. UV/Vis: λ_max_ = 269 nm (∊ = 1.1885 × 10^5^ Lmol^−1^cm^−1^). ^1^H NMR (300 MHz, CDCl_3_): δ = 7.201 (*m*, 4H), 6.682 (*t*, 1H, *J* = 9.6 Hz), 6.501 (*d*, 1H, *J* = 10.5 Hz), 3.056 (*d*, 3H, *J* = 5.4 Hz). ^13^C NMR (300MHz, CDCl_3_): δ = 177, 157, 137, 136, 129, 122, 108, 29.

## Refinement   

Crystal data, data collection and structure refinement details are summarized in Table 2 ▸. Methyl and aromatic hydrogen atoms were placed in geometrically idealized positions (C—H = 0.95–0.98 Å) and constrained to ride on their parent atoms [*U*_iso_(H) = 1.5*U*_eq_(C) and 1.2*U*_eq_(C)], while N—H hydrogens were freely refined.

## Supplementary Material

Crystal structure: contains datablock(s) global, I. DOI: 10.1107/S2056989019009502/pk2619sup1.cif

Supporting information file. DOI: 10.1107/S2056989019009502/pk2619Isup2.cml

CCDC reference: 1937929

Additional supporting information:  crystallographic information; 3D view; checkCIF report

## Figures and Tables

**Table 1:** Hydrogen-bond geometry (Å, °)

*D*—H⋯*A*	*D*—H	H⋯*A*	*D*⋯*A*	*D*—H⋯*A*
N1*A*—H*N*1*A*⋯O1*A*	0.884 (17)	2.099 (16)	2.5453 (16)	110.3 (13)
N1*A*—H*N*1*A*⋯O1*B*	0.884 (17)	2.248 (17)	2.9375 (17)	134.6 (14)
N1*B*—H*N*1*B*⋯O1*B*	0.893 (15)	2.085 (15)	2.5513 (16)	111.5 (12)
N1*B*—H*N*1*B*⋯O1*C*	0.893 (15)	2.385 (15)	3.1566 (18)	144.7 (13)
N1*C*—H*N*1*C*⋯O1*C*	0.890 (16)	2.130 (15)	2.5775 (16)	110.3 (12)
N1*C*—H*N*1*C*⋯O1*A* ^i^	0.890 (16)	2.313 (16)	2.9759 (17)	131.3 (13)
C5*C*—H5*C*⋯O1*B* ^ii^	0.95	2.42	3.2914 (19)	153
C7*B*—H7*B*⋯O1*A*	0.95	2.42	3.3446 (19)	165
C8*C*—H8*C*2⋯O1*A* ^i^	0.98	2.56	3.178 (2)	121

Symmetry codes: (i) 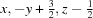; (ii) 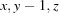.

**Table 2:** Experimental details

Crystal data	
Chemical formula	C_8_H_9_NO
*M*_r_	135.16
Crystal system, space group	Monoclinic, *P*2_1_/*c*
Temperature (K)	100
*a*, *b*, *c* (Å)	17.635 (5), 7.817 (2), 16.718 (4)
β (°)	110.639 (9)
*V* (Å^3^)	2156.8 (10)
*Z*	12
Radiation type	Mo *K*α
μ (mm^−1^)	0.08
Crystal size (mm)	0.58 × 0.30 × 0.28

Data collection	
Diffractometer	Bruker X8 APEXII 4K Kappa CCD
Absorption correction	Multi-scan *SADABS* (Krause *et al.*, 2015 ▸)
*T*_min_, *T*_max_	0.970, 0.977
No. of measured, independent and observed [*I* > 2σ(*I*)] reflections	33879, 5192, 3575
*R*_int_	0.046
(sin θ/λ)_max_ (Å^−1^)	0.660

Refinement	
*R*[*F* ^2^ > 2σ(*F* ^2^)], *wR*(*F* ^2^), *S*	0.039, 0.104, 1.03
No. of reflections	5192
No. of parameters	287
H-atom treatment	H atoms treated by a mixture of independent and constrained refinement
Δρ_max_, Δρ_min_ (e Å^−3^)	0.17, −0.13

Computer programs: *APEX2* and *SAINT-Plus* (Bruker, 2012 ▸), *SHELXS97* (Sheldrick, 2008 ▸), *SHELXL2018* (Sheldrick, 2015 ▸), *DIAMOND* (Brandenburg, 2006 ▸) and *WinGX* (Farrugia, 2012 ▸).

## supplementary crystallographic information

### Crystal data

**Table:**

C_8_H_9_NO	*F*(000) = 864
*M**_r_* = 135.16	*D*_x_ = 1.249 Mg m^−^^3^
Monoclinic, *P*2_1_/*c*	Mo *K*α radiation, λ = 0.71073 Å
Hall symbol: -P 2ybc	Cell parameters from 6617 reflections
*a* = 17.635 (5) Å	θ = 3.5–23.7°
*b* = 7.817 (2) Å	µ = 0.08 mm^−^^1^
*c* = 16.718 (4) Å	*T* = 100 K
β = 110.639 (9)°	Cuboid, yellow
*V* = 2156.8 (10) Å^3^	0.58 × 0.30 × 0.28 mm
*Z* = 12	

### Data collection

**Table:**

Bruker X8 APEXII 4K Kappa CCD diffractometer	5192 independent reflections
Radiation source: fine-focus sealed tube	3575 reflections with *I* > 2σ(*I*)
Graphite monochromator	*R*_int_ = 0.046
ω scans	θ_max_ = 28.0°, θ_min_ = 3.7°
Absorption correction: multi-scan SADABS (Krause *et al.*, 2015)	*h* = −23→23
*T*_min_ = 0.970, *T*_max_ = 0.977	*k* = −10→10
33879 measured reflections	*l* = −20→22

### Refinement

**Table:**

Refinement on *F*^2^	Secondary atom site location: structure-invariant direct methods
Least-squares matrix: full	Hydrogen site location: mixed
*R*[*F*^2^ > 2σ(*F*^2^)] = 0.039	H atoms treated by a mixture of independent and constrained refinement
*wR*(*F*^2^) = 0.104	*w* = 1/[σ^2^(*F*_o_^2^) + (0.0361*P*)^2^ + 0.437*P*] where *P* = (*F*_o_^2^ + 2*F*_c_^2^)/3
*S* = 1.03	(Δ/σ)_max_ < 0.001
5192 reflections	Δρ_max_ = 0.17 e Å^−^^3^
287 parameters	Δρ_min_ = −0.13 e Å^−^^3^
0 restraints	Extinction correction: SHELXL2018 (Sheldrick, 2015)
0 constraints	Extinction coefficient: 0.0113 (9)
Primary atom site location: structure-invariant direct methods	

### Special details

**Table:**

Experimental. The intensity data was collected on a Bruker X8 ApexII 4K Kappa CCD diffractometer using an exposure time of 10 seconds/frame. A total of 1436 frames was collected with a frame width of 0.5° covering up to θ = 27.99° with 99.7% completeness accomplished.
Geometry. All esds (except the esd in the dihedral angle between two l.s. planes) are estimated using the full covariance matrix. The cell esds are taken into account individually in the estimation of esds in distances, angles and torsion angles; correlations between esds in cell parameters are only used when they are defined by crystal symmetry. An approximate (isotropic) treatment of cell esds is used for estimating esds involving l.s. planes.

### Fractional atomic coordinates and isotropic or equivalent isotropic displacement parameters (Å^2^)

**Table:**

	*x*	*y*	*z*	*U*_iso_*/*U*_eq_	
C8A	0.12323 (10)	0.5412 (2)	0.21119 (10)	0.0626 (4)	
H8A1	0.113602	0.421239	0.221702	0.094*	
H8A2	0.172571	0.549845	0.197169	0.094*	
H8A3	0.077051	0.584932	0.163359	0.094*	
C8B	0.49672 (10)	0.4930 (2)	0.26816 (10)	0.0586 (4)	
H8B1	0.530729	0.588791	0.263364	0.088*	
H8B2	0.47058	0.440852	0.2118	0.088*	
H8B3	0.530411	0.407538	0.307747	0.088*	
C8C	0.19718 (11)	0.2656 (2)	−0.10383 (9)	0.0588 (4)	
H8C1	0.234672	0.189118	−0.117954	0.088*	
H8C2	0.187004	0.367098	−0.140513	0.088*	
H8C3	0.145975	0.20569	−0.113027	0.088*	
N1A	0.13282 (7)	0.64142 (16)	0.28726 (8)	0.0460 (3)	
N1B	0.43536 (8)	0.55487 (16)	0.29999 (8)	0.0454 (3)	
N1C	0.23250 (7)	0.31715 (16)	−0.01509 (7)	0.0450 (3)	
O1A	0.17457 (6)	0.83578 (13)	0.41777 (6)	0.0530 (3)	
O1B	0.30974 (5)	0.61826 (14)	0.33715 (6)	0.0515 (3)	
O1C	0.28810 (7)	0.46038 (12)	0.13314 (6)	0.0574 (3)	
HN1A	0.1803 (10)	0.689 (2)	0.3149 (10)	0.064 (5)*	
HN1B	0.3829 (9)	0.5397 (19)	0.2695 (10)	0.052 (4)*	
HN1C	0.2345 (9)	0.428 (2)	−0.0016 (10)	0.056 (5)*	
C1B	0.37461 (8)	0.67009 (17)	0.39220 (8)	0.0379 (3)	
C2B	0.44995 (7)	0.63079 (16)	0.37612 (8)	0.0379 (3)	
C3B	0.52868 (8)	0.66224 (19)	0.43132 (9)	0.0476 (3)	
H3B	0.569782	0.625712	0.410663	0.057*	
C4B	0.55733 (9)	0.7385 (2)	0.51194 (10)	0.0545 (4)	
H4B	0.614666	0.74095	0.538203	0.065*	
C5B	0.51628 (9)	0.8101 (2)	0.55919 (10)	0.0553 (4)	
H5B	0.548086	0.855301	0.613404	0.066*	
C6B	0.43262 (9)	0.82372 (19)	0.53599 (9)	0.0510 (4)	
H6B	0.414495	0.882838	0.575565	0.061*	
C7B	0.37249 (8)	0.76418 (18)	0.46432 (8)	0.0437 (3)	
H7B	0.319314	0.790761	0.462424	0.052*	
C1C	0.27959 (8)	0.30140 (16)	0.13520 (8)	0.0396 (3)	
C2C	0.25275 (7)	0.21032 (16)	0.05204 (8)	0.0346 (3)	
C3C	0.24942 (8)	0.03409 (16)	0.03938 (9)	0.0406 (3)	
H3C	0.236584	−0.000441	−0.018381	0.049*	
C4C	0.26179 (8)	−0.09981 (17)	0.09743 (9)	0.0460 (3)	
H4C	0.257336	−0.210933	0.073189	0.055*	
C5C	0.27941 (9)	−0.09661 (19)	0.18414 (10)	0.0495 (4)	
H5C	0.283417	−0.204059	0.211879	0.059*	
C6C	0.29204 (8)	0.0487 (2)	0.23568 (9)	0.0491 (4)	
H6C	0.301651	0.026655	0.294322	0.059*	
C7C	0.29278 (8)	0.21802 (19)	0.21506 (9)	0.0458 (3)	
H7C	0.304073	0.293672	0.262311	0.055*	
C1A	0.10733 (8)	0.76484 (17)	0.40289 (8)	0.0411 (3)	
C2A	0.07897 (8)	0.65224 (16)	0.32681 (8)	0.0395 (3)	
C3A	0.00556 (8)	0.56527 (19)	0.29566 (10)	0.0538 (4)	
H3A	−0.003564	0.505768	0.243542	0.065*	
C4A	−0.05654 (9)	0.5510 (2)	0.32809 (13)	0.0647 (5)	
H4A	−0.100588	0.48099	0.295409	0.078*	
C5A	−0.06451 (10)	0.6213 (2)	0.39924 (13)	0.0702 (5)	
H5A	−0.112064	0.59234	0.410595	0.084*	
C6A	−0.01009 (11)	0.7312 (3)	0.45709 (12)	0.0707 (5)	
H6A	−0.026039	0.768486	0.502906	0.085*	
C7A	0.06298 (10)	0.7941 (2)	0.45827 (10)	0.0584 (4)	
H7A	0.088461	0.870606	0.504039	0.07*	

### Atomic displacement parameters (Å^2^)

**Table:**

	*U*^11^	*U*^22^	*U*^33^	*U*^12^	*U*^13^	*U*^23^
C8A	0.0635 (10)	0.0735 (11)	0.0479 (9)	−0.0028 (9)	0.0162 (8)	−0.0172 (8)
C8B	0.0646 (10)	0.0627 (10)	0.0585 (10)	0.0095 (8)	0.0343 (8)	0.0034 (8)
C8C	0.0758 (11)	0.0571 (9)	0.0380 (8)	0.0003 (8)	0.0132 (7)	0.0016 (7)
N1A	0.0412 (7)	0.0501 (7)	0.0437 (7)	−0.0052 (6)	0.0114 (5)	−0.0085 (6)
N1B	0.0432 (7)	0.0527 (7)	0.0421 (7)	0.0019 (6)	0.0171 (6)	0.0013 (5)
N1C	0.0587 (7)	0.0373 (6)	0.0384 (6)	−0.0010 (5)	0.0164 (5)	−0.0014 (5)
O1A	0.0484 (6)	0.0480 (6)	0.0582 (6)	−0.0073 (5)	0.0132 (5)	−0.0137 (5)
O1B	0.0353 (5)	0.0712 (7)	0.0441 (6)	−0.0060 (5)	0.0091 (4)	−0.0063 (5)
O1C	0.0861 (8)	0.0361 (5)	0.0494 (6)	−0.0083 (5)	0.0230 (6)	−0.0073 (5)
C1B	0.0360 (7)	0.0408 (7)	0.0354 (7)	−0.0023 (5)	0.0106 (5)	0.0074 (5)
C2B	0.0385 (7)	0.0371 (7)	0.0388 (7)	−0.0007 (5)	0.0146 (6)	0.0094 (6)
C3B	0.0355 (7)	0.0566 (9)	0.0517 (8)	0.0001 (6)	0.0166 (6)	0.0091 (7)
C4B	0.0353 (7)	0.0669 (10)	0.0522 (9)	−0.0082 (7)	0.0042 (6)	0.0085 (8)
C5B	0.0516 (9)	0.0634 (10)	0.0422 (8)	−0.0135 (7)	0.0056 (7)	−0.0022 (7)
C6B	0.0576 (9)	0.0530 (9)	0.0425 (8)	−0.0058 (7)	0.0177 (7)	−0.0046 (7)
C7B	0.0409 (7)	0.0497 (8)	0.0417 (7)	−0.0007 (6)	0.0158 (6)	0.0021 (6)
C1C	0.0402 (7)	0.0381 (7)	0.0416 (7)	−0.0022 (5)	0.0158 (6)	−0.0058 (6)
C2C	0.0327 (6)	0.0361 (6)	0.0365 (7)	0.0003 (5)	0.0141 (5)	−0.0009 (5)
C3C	0.0445 (7)	0.0367 (7)	0.0417 (7)	−0.0003 (6)	0.0164 (6)	−0.0054 (6)
C4C	0.0478 (8)	0.0337 (7)	0.0571 (9)	0.0026 (6)	0.0192 (7)	−0.0010 (6)
C5C	0.0486 (8)	0.0423 (8)	0.0561 (9)	0.0042 (6)	0.0165 (7)	0.0120 (7)
C6C	0.0462 (8)	0.0590 (9)	0.0393 (8)	−0.0020 (7)	0.0117 (6)	0.0074 (7)
C7C	0.0492 (8)	0.0502 (8)	0.0372 (7)	−0.0054 (6)	0.0143 (6)	−0.0050 (6)
C1A	0.0396 (7)	0.0367 (7)	0.0420 (7)	0.0057 (6)	0.0080 (6)	0.0033 (6)
C2A	0.0368 (7)	0.0347 (7)	0.0417 (7)	0.0039 (5)	0.0072 (6)	0.0032 (6)
C3A	0.0406 (8)	0.0510 (8)	0.0620 (10)	−0.0038 (6)	0.0086 (7)	−0.0062 (7)
C4A	0.0392 (8)	0.0596 (10)	0.0907 (13)	−0.0038 (7)	0.0170 (8)	0.0064 (9)
C5A	0.0445 (9)	0.0794 (12)	0.0920 (14)	0.0088 (9)	0.0308 (9)	0.0247 (11)
C6A	0.0661 (11)	0.0884 (13)	0.0683 (11)	0.0241 (10)	0.0372 (10)	0.0169 (10)
C7A	0.0603 (10)	0.0643 (10)	0.0504 (9)	0.0113 (8)	0.0194 (8)	−0.0021 (8)

### Geometric parameters (Å, º)

**Table:**

C8A—N1A	1.4518 (19)	C4B—H4B	0.95
C8A—H8A1	0.98	C5B—C6B	1.391 (2)
C8A—H8A2	0.98	C5B—H5B	0.95
C8A—H8A3	0.98	C6B—C7B	1.3716 (19)
C8B—N1B	1.4470 (18)	C6B—H6B	0.95
C8B—H8B1	0.98	C7B—H7B	0.95
C8B—H8B2	0.98	C1C—C7C	1.4293 (19)
C8B—H8B3	0.98	C1C—C2C	1.4832 (18)
C8C—N1C	1.4492 (18)	C2C—C3C	1.3919 (18)
C8C—H8C1	0.98	C3C—C4C	1.3913 (19)
C8C—H8C2	0.98	C3C—H3C	0.95
C8C—H8C3	0.98	C4C—C5C	1.372 (2)
N1A—C2A	1.3376 (18)	C4C—H4C	0.95
N1A—HN1A	0.884 (17)	C5C—C6C	1.395 (2)
N1B—C2B	1.3444 (17)	C5C—H5C	0.95
N1B—HN1B	0.893 (15)	C6C—C7C	1.369 (2)
N1C—C2C	1.3423 (16)	C6C—H6C	0.95
N1C—HN1C	0.890 (16)	C7C—H7C	0.95
O1A—O1A	0.000 (3)	C1A—C7A	1.426 (2)
O1A—C1A	1.2519 (16)	C1A—C2A	1.4812 (19)
O1B—O1B	0.0000 (19)	C2A—C3A	1.3906 (19)
O1B—C1B	1.2561 (15)	C3A—C4A	1.387 (2)
O1C—O1C	0.000 (3)	C3A—H3A	0.95
O1C—C1C	1.2537 (16)	C4A—C5A	1.362 (3)
C1B—C7B	1.4240 (19)	C4A—H4A	0.95
C1B—C2B	1.4777 (18)	C5A—C6A	1.393 (3)
C2B—C3B	1.3917 (19)	C5A—H5A	0.95
C3B—C4B	1.395 (2)	C6A—C7A	1.373 (2)
C3B—H3B	0.95	C6A—H6A	0.95
C4B—C5B	1.366 (2)	C7A—H7A	0.95
			
N1A—C8A—H8A1	109.5	C6B—C7B—C1B	132.22 (14)
N1A—C8A—H8A2	109.5	C6B—C7B—H7B	113.9
H8A1—C8A—H8A2	109.5	C1B—C7B—H7B	113.9
N1A—C8A—H8A3	109.5	O1C—C1C—O1C	0.00 (10)
H8A1—C8A—H8A3	109.5	O1C—C1C—C7C	119.73 (12)
H8A2—C8A—H8A3	109.5	O1C—C1C—C7C	119.73 (12)
N1B—C8B—H8B1	109.5	O1C—C1C—C2C	116.86 (12)
N1B—C8B—H8B2	109.5	O1C—C1C—C2C	116.86 (12)
H8B1—C8B—H8B2	109.5	C7C—C1C—C2C	123.36 (12)
N1B—C8B—H8B3	109.5	N1C—C2C—C3C	120.28 (12)
H8B1—C8B—H8B3	109.5	N1C—C2C—C1C	112.83 (11)
H8B2—C8B—H8B3	109.5	C3C—C2C—C1C	126.87 (12)
N1C—C8C—H8C1	109.5	C4C—C3C—C2C	130.60 (13)
N1C—C8C—H8C2	109.5	C4C—C3C—H3C	114.7
H8C1—C8C—H8C2	109.5	C2C—C3C—H3C	114.7
N1C—C8C—H8C3	109.5	C5C—C4C—C3C	130.16 (13)
H8C1—C8C—H8C3	109.5	C5C—C4C—H4C	114.9
H8C2—C8C—H8C3	109.5	C3C—C4C—H4C	114.9
C2A—N1A—C8A	125.69 (13)	C4C—C5C—C6C	126.51 (13)
C2A—N1A—HN1A	115.0 (11)	C4C—C5C—H5C	116.7
C8A—N1A—HN1A	118.8 (11)	C6C—C5C—H5C	116.7
C2B—N1B—C8B	125.27 (13)	C7C—C6C—C5C	130.18 (14)
C2B—N1B—HN1B	114.4 (10)	C7C—C6C—H6C	114.9
C8B—N1B—HN1B	120.3 (10)	C5C—C6C—H6C	114.9
C2C—N1C—C8C	125.07 (12)	C6C—C7C—C1C	131.55 (13)
C2C—N1C—HN1C	114.7 (10)	C6C—C7C—H7C	114.2
C8C—N1C—HN1C	119.5 (10)	C1C—C7C—H7C	114.2
O1A—O1A—C1A	0 (10)	O1A—C1A—O1A	0.00 (9)
O1B—O1B—C1B	0 (10)	O1A—C1A—C7A	119.86 (13)
O1C—O1C—C1C	0 (10)	O1A—C1A—C7A	119.86 (13)
O1B—C1B—O1B	0.00 (15)	O1A—C1A—C2A	116.32 (12)
O1B—C1B—C7B	119.87 (12)	O1A—C1A—C2A	116.32 (12)
O1B—C1B—C7B	119.87 (12)	C7A—C1A—C2A	123.82 (13)
O1B—C1B—C2B	116.40 (12)	N1A—C2A—C3A	120.88 (13)
O1B—C1B—C2B	116.40 (12)	N1A—C2A—C1A	112.23 (11)
C7B—C1B—C2B	123.72 (12)	C3A—C2A—C1A	126.88 (13)
N1B—C2B—C3B	121.26 (12)	C4A—C3A—C2A	130.67 (16)
N1B—C2B—C1B	112.32 (11)	C4A—C3A—H3A	114.7
C3B—C2B—C1B	126.40 (13)	C2A—C3A—H3A	114.7
C2B—C3B—C4B	130.76 (14)	C5A—C4A—C3A	130.39 (16)
C2B—C3B—H3B	114.6	C5A—C4A—H4A	114.8
C4B—C3B—H3B	114.6	C3A—C4A—H4A	114.8
C5B—C4B—C3B	130.44 (14)	C4A—C5A—C6A	126.67 (16)
C5B—C4B—H4B	114.8	C4A—C5A—H5A	116.7
C3B—C4B—H4B	114.8	C6A—C5A—H5A	116.7
C4B—C5B—C6B	126.62 (14)	C7A—C6A—C5A	130.12 (17)
C4B—C5B—H5B	116.7	C7A—C6A—H6A	114.9
C6B—C5B—H5B	116.7	C5A—C6A—H6A	114.9
C7B—C6B—C5B	129.43 (15)	C6A—C7A—C1A	131.32 (16)
C7B—C6B—H6B	115.3	C6A—C7A—H7A	114.3
C5B—C6B—H6B	115.3	C1A—C7A—H7A	114.3
			
O1B—O1B—C1B—C7B	0.00 (7)	N1C—C2C—C3C—C4C	−174.98 (13)
O1B—O1B—C1B—C2B	0.00 (11)	C1C—C2C—C3C—C4C	7.0 (2)
C8B—N1B—C2B—C3B	2.3 (2)	C2C—C3C—C4C—C5C	1.4 (3)
C8B—N1B—C2B—C1B	−176.57 (13)	C3C—C4C—C5C—C6C	−2.5 (3)
O1B—C1B—C2B—N1B	4.66 (16)	C4C—C5C—C6C—C7C	−2.3 (3)
O1B—C1B—C2B—N1B	4.66 (16)	C5C—C6C—C7C—C1C	1.4 (3)
C7B—C1B—C2B—N1B	−174.08 (12)	O1C—C1C—C7C—C6C	−175.92 (15)
O1B—C1B—C2B—C3B	−174.13 (13)	O1C—C1C—C7C—C6C	−175.92 (15)
O1B—C1B—C2B—C3B	−174.13 (13)	C2C—C1C—C7C—C6C	6.6 (2)
C7B—C1B—C2B—C3B	7.1 (2)	O1A—O1A—C1A—C7A	0.00 (3)
N1B—C2B—C3B—C4B	179.84 (15)	O1A—O1A—C1A—C2A	0.00 (2)
C1B—C2B—C3B—C4B	−1.5 (2)	C8A—N1A—C2A—C3A	−0.8 (2)
C2B—C3B—C4B—C5B	−2.6 (3)	C8A—N1A—C2A—C1A	179.80 (13)
C3B—C4B—C5B—C6B	−0.1 (3)	O1A—C1A—C2A—N1A	1.88 (17)
C4B—C5B—C6B—C7B	3.1 (3)	O1A—C1A—C2A—N1A	1.88 (17)
C5B—C6B—C7B—C1B	0.8 (3)	C7A—C1A—C2A—N1A	−177.78 (13)
O1B—C1B—C7B—C6B	174.25 (15)	O1A—C1A—C2A—C3A	−177.44 (13)
O1B—C1B—C7B—C6B	174.25 (15)	O1A—C1A—C2A—C3A	−177.44 (13)
C2B—C1B—C7B—C6B	−7.1 (2)	C7A—C1A—C2A—C3A	2.9 (2)
O1C—O1C—C1C—C7C	0.0 (2)	N1A—C2A—C3A—C4A	176.30 (16)
O1C—O1C—C1C—C2C	0.0 (2)	C1A—C2A—C3A—C4A	−4.4 (3)
C8C—N1C—C2C—C3C	7.7 (2)	C2A—C3A—C4A—C5A	1.6 (3)
C8C—N1C—C2C—C1C	−174.00 (13)	C3A—C4A—C5A—C6A	1.5 (3)
O1C—C1C—C2C—N1C	−7.16 (17)	C4A—C5A—C6A—C7A	−0.6 (3)
O1C—C1C—C2C—N1C	−7.16 (17)	C5A—C6A—C7A—C1A	−1.8 (3)
C7C—C1C—C2C—N1C	170.43 (12)	O1A—C1A—C7A—C6A	−178.88 (16)
O1C—C1C—C2C—C3C	171.01 (13)	O1A—C1A—C7A—C6A	−178.88 (16)
O1C—C1C—C2C—C3C	171.01 (13)	C2A—C1A—C7A—C6A	0.8 (3)
C7C—C1C—C2C—C3C	−11.4 (2)		

### Hydrogen-bond geometry (Å, º)

**Table:**

*D*—H···*A*	*D*—H	H···*A*	*D*···*A*	*D*—H···*A*
N1*A*—H*N*1*A*···O1*A*	0.884 (17)	2.099 (16)	2.5453 (16)	110.3 (13)
N1*A*—H*N*1*A*···O1*B*	0.884 (17)	2.248 (17)	2.9375 (17)	134.6 (14)
N1*B*—H*N*1*B*···O1*B*	0.893 (15)	2.085 (15)	2.5513 (16)	111.5 (12)
N1*B*—H*N*1*B*···O1*C*	0.893 (15)	2.385 (15)	3.1566 (18)	144.7 (13)
N1*C*—H*N*1*C*···O1*C*	0.890 (16)	2.130 (15)	2.5775 (16)	110.3 (12)
N1*C*—H*N*1*C*···O1*A*^i^	0.890 (16)	2.313 (16)	2.9759 (17)	131.3 (13)
C5*C*—H5*C*···O1*B*^ii^	0.95	2.42	3.2914 (19)	153
C7*B*—H7*B*···O1*A*	0.95	2.42	3.3446 (19)	165
C8*C*—H8*C*2···O1*A*^i^	0.98	2.56	3.178 (2)	121

Symmetry codes: (i) *x*, −*y*+3/2, *z*−1/2; (ii) *x*, *y*−1, *z*.

## References

[bb1] Barret, M., Bhatia, P., Kociok-Köhn, G. & Molloy, K. (2014). *Transition Met. Chem.* **39**, 543–551.

[bb2] Bhalla, G., Oxgaard, J., Goddard, W. & Periana, R. (2005). *Organometallics*, **24**, 3229–3232.

[bb3] Boschi, A., Martini, P., Janevik-Ivanovska, E. & Duatti, A. (2018). *Drug Discovery Today*, **23**, 1489–1501. 10.1016/j.drudis.2018.04.002 29635027

[bb4] Brandenburg, K. (2006). *DIAMOND*. Crystal Impact GbR, Bonn, Germany.

[bb5] Bruker (2012). *APEX2*, *SAINT*, Bruker AXS Inc, Madison, Wisconsin, USA.

[bb6] Crous, R., Datt, M., Foster, D., Bennie, L., Steenkamp, C., Huyser, J., Kirsten, L., Steyl, G. & Roodt, A. (2005). *Dalton Trans.* pp. 1108–1116. 10.1039/b416917d 15739014

[bb7] Dias, H. V. R., Jin, W. & Ratcliff, R. E. (1995). *Inorg. Chem.* **34**, 6100–6105.

[bb8] Dwivedi, A., Binnani, C., Tyagi, D., Rawat, K., Li, P., Zhao, Y., Mobin, S. M., Pathak, B. & Singh, S. K. (2016). *Inorg. Chem.* **55**, 6739–6749. 10.1021/acs.inorgchem.6b01028 27305143

[bb9] Farrugia, L. J. (2012). *J. Appl. Cryst.* **45**, 849–854.

[bb10] Green, M. & Welch, M. (1989). *Int. J. Radiat. Appl. Instrum. B*, **16**, 435–448. 10.1016/0883-2897(89)90053-6 2681083

[bb11] Groom, C. R., Bruno, I. J., Lightfoot, M. P. & Ward, S. C. (2016). *Acta Cryst.* B**72**, 171–179. 10.1107/S2052520616003954 PMC4822653 27048719

[bb12] Guo, H., Roman, D. & Beemelmanns, C. (2019). *Natural Product Reports.* https://doi.org/10.1039/C8NP00078F. 10.1039/c8np00078f 30556819

[bb13] Hsiao, C. J., Hsiao, S. H., Chen, W. L., Guh, J. H., Hsiao, G., Chan, Y. J., Lee, T. H. & Chung, C. L. (2012). *Chem. Biol. Interact.* **197**, 23–30. 10.1016/j.cbi.2012.03.004 22450442

[bb14] Krause, L., Herbst-Irmer, R., Sheldrick, G. M. & Stalke, D. (2015). *J. Appl. Cryst.* **48**, 3–10. 10.1107/S1600576714022985 PMC4453166 26089746

[bb15] Liu, S. & Yamauchi, H. (2006). *Biochem. Biophys. Res. Commun.* **351**, 26–32. 10.1016/j.bbrc.2006.09.166 17055455

[bb16] Manicum, A., Schutte-Smith, M., Alexander, O., Twigge, L., Roodt, A. & Visser, H. (2019). *Inorg. Chem. Commun.* **101**, 93–98.

[bb17] Manicum, A., Schutte-Smith, M. & Visser, H. (2018). *Polyhedron*, **145**, 80–87.

[bb18] Markovitch, O. & Agmon, N. (2008). *Mol. Phys.* **106**, 485–495.

[bb19] Nepveu, F., Jasanada, F. & Walz, L. (1993). *Inorg. Chim. Acta*, **211**, 141–147.

[bb20] Nishinaga, T., Aono, T., Isomura, E., Watanabe, S., Miyake, Y., Miyazaki, A., Enoki, T., Miyasaka, H., Otani, H. & Iyoda, M. (2010). *Dalton Trans.* **39**, 2293–2300. 10.1039/b912255a 20162203

[bb21] Nozoe, T., Lin, L. C., Hsu, C., Tsay, S., Hakimelahib, G. H. & Hwu, J. R. (1997). *J. Chem. Res. (S)*, pp. 362–363.

[bb22] Ononye, S. N., VanHeyst, M. D., Oblak, E. Z., Zhou, W., Ammar, M., Anderson, A. C. & Wright, D. L. (2013). *ACS Med. Chem. Lett.* **4**, 757–761. 10.1021/ml400158k PMC4027479 24900743

[bb23] Roesky, P. & Bürgstein, M. (1999). *Inorg. Chem.* **38**, 5629–5632. 10.1021/ic990533c 11671293

[bb24] Roesky, P. W. (2000). *Chem. Soc. Rev.* **29**, 335–345.

[bb25] Roodt, A., Otto, S. & Steyl, G. (2003). *Coord. Chem. Rev.* **245**, 121–137.

[bb26] Saleh, N. A., Zfiefak, A., Mordarski, M. & Pulverer, G. (1988). *Zentralbl. Bakteriol. MikroBiol. Hyg. Med. Microbiol. Infect. Dis. Virol. Paras.* **270**, 160–170. 10.1016/s0176-6724(88)80153-6 3223137

[bb27] Schutte, M., Kemp, G., Visser, H. & Roodt, A. (2011). *Inorg. Chem.* **50**, 12486–12498. 10.1021/ic2013792 22111710

[bb28] Schutte, M., Roodt, A. & Visser, H. (2012). *Inorg. Chem.* **51**, 11996–12006. 10.1021/ic301891u 23088314

[bb29] Schutte, M., Visser, H. G. & Roodt, A. (2010). *Acta Cryst.* E**66**, m859–m860. 10.1107/S1600536810024505 PMC3006975 21587765

[bb30] Schutte-Smith, M., Roodt, A. & Visser, H. G. (2019). *Dalton Trans.* https://doi.org/10.1039/C9DT01528K.

[bb31] Sheldrick, G. M. (2008). *Acta Cryst.* A**64**, 112–122. 10.1107/S0108767307043930 18156677

[bb32] Sheldrick, G. M. (2015). *Acta Cryst.* C**71**, 3–8.

[bb33] Shimanouchi, H. & Sasada, Y. (1973). *Acta Cryst.* B**29**, 81–90.

[bb34] Siwatch, R. K., Kundu, S., Kumar, S. & Nagendran, S. (2011). *Organometallics*, **30**, 1998–2005.

[bb35] Steyl, G., Muller, T. J. & Roodt, A. (2010). *Acta Cryst.* E**66**, m1508. 10.1107/S1600536810043503 PMC3011584 21589214

[bb36] Tavis, J. E. & Lomonosova, E. (2015). *Antiviral Res.* **118**, 132–138. 10.1016/j.antiviral.2015.04.002 PMC4424167 25862291

[bb37] Wong, V., Po, C., Leung, S., Chan, A., Yang, S., Zhu, B., Cui, X. & Yam, V. W. (2018). *J. Am. Chem. Soc.* **140**, 657–666. 10.1021/jacs.7b09770 29303262

